# Screening of the Antimicrobial Activity against Drug Resistant Bacteria of *Photorhabdus* and *Xenorhabdus* Associated with Entomopathogenic Nematodes from Mae Wong National Park, Thailand

**DOI:** 10.3389/fmicb.2017.01142

**Published:** 2017-06-28

**Authors:** Paramaporn Muangpat, Temsiri Yooyangket, Chamaiporn Fukruksa, Manawat Suwannaroj, Thatcha Yimthin, Sutthirat Sitthisak, Narisara Chantratita, Apichat Vitta, Nicholas J. Tobias, Helge B. Bode, Aunchalee Thanwisai

**Affiliations:** ^1^Department of Microbiology and Parasitology, Faculty of Medical Science, Naresuan UniversityPhitsanulok, Thailand; ^2^Department of Microbiology and Immunology, Faculty of Tropical Medicine, Mahidol UniversityBangkok, Thailand; ^3^Centre of Excellence in Medical Biotechnology, Faculty of Medical Science, Naresuan UniversityPhitsanulok, Thailand; ^4^Center of Excellence for Biodiversity, Faculty of Sciences, Naresuan UniversityPhitsanulok, Thailand; ^5^Merck-Stiftungsprofessur für Molekulare Biotechnologie, Fachbereich Biowissenschaften, Goethe Universität FrankfurtFrankfurt am Main, Germany; ^6^Buchmann Institute for Molecular Life Sciences, Goethe University FrankfurtFrankfurt am Main, Germany

**Keywords:** *Xenorhabdus*, *Photorhabdus*, entomopathogenic nematodes, antimicrobial activity, drug-resistant bacteria

## Abstract

*Photorhabdus* and *Xenorhabdus* are symbiotic with entomopathogenic nematodes (EPNs) of the genera *Heterorhabditis* and *Steinernema*, respectively. These bacteria produce several secondary metabolites including antimicrobial compounds. The objectives of this study were to isolate and identify EPNs and their symbiotic bacteria from Mae Wong National Park, Thailand and to evaluate the antibacterial activities of symbiont extracts against drug resistant bacteria. A total of 550 soil samples from 110 sites were collected between August 2014 and July 2015. A total of EPN isolates were obtained through baiting and White trap methods, which yielded 21 *Heterorhabditis* and 3 *Steinernema* isolates. Based on molecular identification and phylogenetic analysis, the most common species found in the present study was *P. luminescens* subsp. *akhurstii* associated with *H. indica*. Notably, two species of EPNs, *H. zealandica* and *S. kushidai*, and two species of symbiotic bacteria, *X. japonica* and *P. temperata* subsp. *temperata* represented new recorded organisms in Thailand. Furthermore, the association between *P. temperata* subsp. *temperata* and *H. zealandica* has not previously been reported worldwide. Disk diffusion, minimal inhibitory concentration, and minimal bactericidal concentration analyses demonstrated that the crude compound extracted by ethyl acetate from *P. temperata* subsp. *temperata* could inhibit the growth of up to 10 strains of drug resistant bacteria. Based on HPLC-MS analysis, compound classes in bacterial extracts were identified as GameXPeptide, xenoamicin, xenocoumacin, mevalagmapeptide phurealipids derivatives, and isopropylstilbene. Together, the results of this study provide evidence for the diversity of EPNs and their symbiotic bacteria in Mae Wong National Park, Thailand and demonstrate their novel associations. These findings also provide an important foundation for further research regarding the antimicrobial activity of *Photorhabdus* bacteria.

## Introduction

*Photorhabdus* and *Xenorhabdus* are symbiotic with entomopathogenic nematodes (EPNs) of the genera *Heterorhabditis* and *Steinernema*, respectively (Hominick, 2002). The infective juvenile stages (IJs) of EPNs are distributed within natural and agricultural soils worldwide and are successfully utilized for the biological control of insect pests (Hominick, 2002). *Photorhabdus* and *Xenorhabdus* belong to Family Enterobacteriaceae, which are Gram negative, rod shaped, and motile via peritrichous flagella. These bacteria are carried in the intestines of IJs that invade into the hemocoel of an insect host through the mouth, spiracles, and anus whereupon they are released from the IJ intestines into the hemolymph (Wang and Gaugler, 1998). The complex between bacterium and nematode destroys the immune response of the larval insect host and leads to its death it within 24–48 h. Consequently, the body of insect cadaver exhibits a red, beige, or black color and slow putrefaction. The EPNs develop through one to three generations in the host cadaver by feeding on the bacteria and dead insect tissue. Upon depletion of the food resources, the IJs emerge from the host cadaver to search for a new host (Wang and Gaugler, 1998).

Notably, *Photorhabdus* and *Xenorhabdus* can produce several secondary metabolites including insecticidal and antimicrobial compounds such as benzylideneacetone, phenethylamines, indole, xenocoumacins and 3,5-dihydroxy-4-isopropylstilbene (McInerney et al., 1991; Eleftherianos et al., 2007). Several studies on the bioactive compounds of *Xenorhabdus* and *Photorhabdus* spp. against various microorganisms have demonstrated their antibacterial (Akhurst, 1982), antimicrobial (Genhui, 1996), and antiparasitic activities (Grundmann et al., 2014).

*Photorhabdus* and *Xenorhabdus* have been reported from all continents of the world except Antarctica, with approximately 30 species (Hominick, 2002; Tailliez et al., 2010; Ferreira et al., 2013a,b, 2014). In turn, worldwide descriptions of EPNs include approximately 26 species of *Heterorhabditis* and 100 species of *Steinernema* (Adams et al., 2006; Li et al., 2012; Thanwisai et al., 2012; Cimen et al., 2014, 2015; Malan et al., 2014; Nthenga et al., 2014; Phan et al., 2014; Vitta et al., 2015). In Thailand, at least two species of *Xenorhabdus* including *X. stockiae* and *X. miraniensis* have been described (Tailliez et al., 2010; Thanwisai et al., 2012) as well as two species with five subspecies of *Photorhabdus*; i.e., *P. luminescens* subsp. *akhurstii*, *P. luminescens* subsp. *hainanensis*, *P. luminescens* subsp. *laumondii*, *P. asymbiotica* subsp. *australis*, and *P. luminescens* subsp. *namnaonensis* from across the country (Maneesakorn et al., 2011; Thanwisai et al., 2012; Glaeser et al., 2017). In addition, nine species of EPNs including *S. siamkayai*, *S. minutum*, *S. websteri*, *S. khoisanae*, *S. scarabaei*, *H. indica*, *H. bacteriophora*, *H. baujardi*, and *H. gerrardi* have been reported across the country (Stock, 1998; Maneesakorn et al., 2010, 2011; Thanwisai et al., 2012; Vitta et al., 2015, 2017). Distributions of EPNs and their bacterial symbionts can be influenced by climate and other ecological communities. The majority of soil locations from Thailand that EPNs have previously been isolated comprise roadside verge, fruit crops, rice fields, and the banks of rivers and ponds (Tangchitsomkid and Sontirat, 1998; Thanwisai et al., 2012; Vitta et al., 2015); however, there are no reports regarding the survey of EPNs and their symbiotic bacteria in the National Park of Thailand.

The main objective of our study was to isolate and identify EPNs and their symbiotic bacteria, *Xenorhabdus* and *Photorhabdus*, from Mae Wong National Park, Kamphaeng Phet Province, Thailand. We also evaluated the antibacterial activities of identified *Xenorhabdus* and *Photorhabdus* extracts against drug resistant bacteria using the disk diffusion method as well as minimal inhibitory concentration (MIC) and minimal bactericidal concentration (MBC) analyses.

## Materials and Methods

### Soil Sampling

The soil collection procedures in Mae Wong Nation Park were approved and permitted by the Department of National Parks, Wildlife and Plant Conservation, Thailand (Permission number 0907.4/7432). A total of 550 soil samples were randomly collected from 110 different sites in Mae Wong National Park, Kamphaeng Phet Province, Thailand. Soil samples were collected between August 2014 and July 2015. The process for soil collection followed Thanwisai et al. (2012). Site locations, soil temperatures, pH, and moisture were recorded. The samples were transported to the Department of Microbiology and Parasitology, Faculty of Medical Science, Naresuan University, Phitsanulok Province, Thailand for isolation of EPNs.

### Isolation and Identification of EPNs

Entomopathogenic nematodes were obtained from soil samples using the baiting technique with *Galleria mellonella* larvae (Bedding and Akhurst, 1975). After 5 days as bait, the dead *G. mellonella* were removed from the soil and were subsequently placed on White traps (White, 1927), in which the emerging IJs were then collected and stored at -20°C for further DNA extraction. The IJs (200–300 nematodes) from each isolate were used for DNA extraction as described (Thanwisai et al., 2012). To identify species of EPNs, polymerase chain reaction (PCR) was performed using the primers TW81_F (5′-GTT TCC GTA GGT GAA CCT GC-3′) and AB28_R (5′-ATA TGC TTA AGT TCA GCG GGT-3′) to amplify a region of the internal transcribed spacer locus (ITS) for *Heterorhabditis* as well as 539_F (5′- GGA TTT CCT TAG TAA CTG CGA GTG-3′) and 535_R (5′-TAG TCT TTC GCC CCT ATA CCC TT-3′) to amplify a region of 28S rDNA for *Steinernema* (Stock et al., 2001). The PCR components (30 μl) consisted of 7.5 μl DNA extracted solution, 0.6 μl dNTPs (200 μM), 1.2 μl each primer (5 μM), 4.2 μl MgCl2 (25 mM), 3 μl 10X buffer, 0.3 μl 5 U/ml Tag polymerase, and 12 μl distilled water. The cycling conditions for ITS were as follows: 1 cycle of 95°C for 5 min followed by 35 cycles of 94°C for 1 min, 50°C for 30 s, 72°C for 1 min, and a final extension at 72°C for 7 min. The cycling conditions for 28S rDNA were as follows: 1 cycle of 95°C for 5 min followed by 35 cycles of 94°C for 1 min, 55°C for 30 s, 72°C for 45 s, and a final extension at 72°C for 7 min. Both reactions were performed in the Applied Biosystems thermal cycler (Applied BiosystemsTM VeritiTM thermal cycler, Pittsburgh, PA, United States). PCR products were checked by electrophoresis over 30 min, 100 V on a 1.2% TBE-buffered agarose gel stained with ethidium bromide. PCR products were purified using a NucleoSpin Gel and PCR Clean-up column (Macerey-Nagel Ltd., Düren, Germany) as recommended by the manufacturer. The purified PCR products were sequenced at Macrogen Inc. Service (Seoul, Korea).

### Isolation and Identification of *Photorhabdus* and *Xenorhabdus*

*Photorhabdus* and *Xenorhabdus* were isolated from the hemolymph of the dead *G. mellonella* infected with EPNs. Hemolymph was streaked on nutrient bromothymol blue triphenyltetrazolium chloride agar (NBTA) and incubated at room temperature in the dark for 4 days. Green and blue colonies were sub-cultured for further genomic DNA extractions. A single colony was then transferred to a 15 ml centrifuge tube containing 3 ml Luria-Bertani (LB) broth and shaken at 180 rpm for 18–24 h. Genomic DNA of each bacterial isolate was extracted from 3 ml culture using a genomic DNA mini kit (Blood/culture cell; Geneaid Biotech Ltd., New Taipei, Taiwan) as recommend by the manufacturer.

To identify *Xenorhabdus* and *Photorhabdus*, the Recombinase A gene (*rec*A) was amplified using primers *rec*A_F (5′-GCT ATT GAT GAA AAT AAA CA-3′) and *rec*A_R (5′-RAT TTT RTC WCC RTT RTA GCT-3′) (Tailliez et al., 2010). The PCR reagents was mixed in a final volume of 30 μl, which consisted of 3 μl DNA extract, 0.6 μl dNTPs (200 μM), 1.2 μl each primer (5 μM), 4.2 μl MgCl_2_ (25 mM), 3 μl 10X reaction buffer, and 0.3 μl 5 U/ml Taq polymerase plus 12 μl distilled water. The cycling conditions for *Xenorhabdus* were as follows: 1 cycle of 94°C for 5 min followed by 30 cycles of 94°C for 1 min, 50°C for 1 min, 72°C for 2 min, and a final extension at 72°C for 7 min. The cycling conditions for *Photorhabdus* were as follows: 1 cycle of 94°C for 5 min followed by 30 cycles of 94°C for 1 min, 50°C for 45 s, 72°C for 1.30 min, and a final extension at 72°C for 7 min. PCR product analysis, purification and sequencing were performed as for EPN characterization.

### Phylogenetic Analysis

Sequence assembly and editing were performed using SeqmanII DNASTAR Inc., (Madison, WI, United States). Sequences were aligned using Clustal W (Thompson et al., 1994) and compared with sequences from other known species in GenBank using the BLASTN algorithm. Phylogenetic analyses of sequence data were performed using MEGA 6.0 (Tamura et al., 2013). A bootstrap consensus tree was inferred from 1000 replicates.

### Preparation of Drug Resistant Bacteria

We used 12 strains of drug-resistant bacteria including *Acinetobacter baumannii* (three clinical strains), *Escherichia coli* (one clinical strain), *E. coli* ATCC^®^ 35218 (one strain), *Klebsiella pneumoniae* ATCC^®^ 700603 (one strain), *Enterococcus faecalis* ATCC^®^ 51299 (one strain), *Pseudomonas aeruginosa* (one clinical strain), *P. aeruginosa* ATCC^®^ 27853 (one strain), *Staphylococcus aureus* (two clinical isolates), and *S. aureus* ATCC^®^ 20475 (one strain) for screening and assessment using the disk diffusion method. The clinical isolates of *A. baumannii*, *E. coli*, *P. aeruginosa*, and *S. aureus* were obtained from patients with positive samples processed by the diagnostic microbiology laboratory at a hospital in Thailand between February 2011 and December 2014 (Supplementary Table S1). Speciation of bacteria were determined using Standard Operating Procedures supplied by the Department of Medical Science, Ministry of Public Heath, Thailand and Clinical and Laboratory Standards Institute (CLSI) guidelines (M100-S24 and M100-S25), respectively. Each bacterial strain was streaked on Mueller Hinton agar (MHA) and incubated at 37°C for 24 h. A single colony was picked, dissolved in phosphate buffered saline solution, and the concentration adjusted to 0.08–0.13 (0.5 McFarland standard) (Seier-Petersen et al., 2014) according to spectrophotometric measurement at a wavelength of 600 nm.

### Preparation of Whole Cell Suspension and Cell-Free Supernatant

We utilized 24 isolates of symbiotic bacteria including 20 isolates of *P. luminescens* subsp. *akhurstii*, 2 isolates of *X. stockiae*, 1 isolate of *X. Japonica*, and 1 isolate of *P. temperata* subsp. *temperata* for screening. A bacterial stock of each isolate was streaked on NBTA medium and incubated at room temperature in the dark for 4 days. A single colony was transferred and grown in 25 ml LB broth and shaken at 180 rpm for 24 h. The concentration of the whole cell suspension was adjusted to 10^7^–10^8^ CFU/ml using phosphate buffered saline solution. To prepare cell-free supernatant, 1 ml whole cell suspension was centrifuged at 12,074 *g* (Centurion, United States) for 5 min. The supernatant was then filtered using a 0.22-μm filter. The flow-through was used as the cell-free supernatant (Bussaman et al., 2012).

### Screening of *Photorhabdus* and *Xenorhabdus* Isolates

To screen *Xenorhabdus* (3 isolates) and *Photorhabdus* (21 isolates) against drug resistant bacteria, 20 μl whole cell suspension and of cell-free supernatant were dropped onto a sterile 6-mm paper disk. The disk was then allowed to dry for 3–5 min and transferred onto the surface of MHA plates that had been plated with 12 strains of drug resistant bacteria. Antibiotic disks were used as positive controls. The MHA plates were incubated at 37°C for 24 h. The diameter of the inhibition zone was measured in millimeter units.

### Bacterial Extracts

*Xenorhabdus* (two isolates) and *Photorhabdus* (nine isolates) that could inhibit the growth of at least one strain of drug resistant bacteria during screening were then subcultured on NBTA medium for further extraction of crude compounds. The plates were incubated at room temperature in the dark for 4 days. A single colony of each isolate was transferred and grown by shaking in a 1000-ml flask containing 500 ml LB at 180 rpm for 72 h. To extract the crude bioactive compound, 1000 ml ethyl acetate was added to the culture and mixed well. The flask was then allowed to stand at room temperature for 24 h. The extraction from each isolate was performed three times to maximize the level of crude compound. All bacterial extracts were concentrated using a rotary vacuum evaporator (Buchi, Flawil, Switzerland). The condensed extracts of all bacterial isolates were weighted and stored at -20°C until used.

### Disk Diffusion Method

Bacterial extracts from each isolate were dissolved in 1 ml dimethyl sulfoxide (DMSO) Then, 10 μl solution was dropped onto a sterile 6-mm disk, which was placed on the MHA plated with drug resistant bacteria. The plates were then incubated at 37°C for 24 h. Antibiotic disks and DMSO were used as a positive and negative control, respectively. The diameter in millimeter of the clear zone was measured using a ruler.

### Minimal Inhibitory Concentrations (MIC**)**

The MIC of nine bacterial extracts was determined using the broth twofold serial dilution method in 96-well micro titer plates. Initial concentrations of all *P. luminescens* and *P. temperata* extracts were dissolved in DMSO to generate 500 and 220 mg/ml solutions, respectively. Then, 100 μl of each extract was mixed with 100 μl MH broth in a well of the micro titer plates followed by twofold serial dilution. To each well, 100 μl bacterial suspension (1 × 10^8^ cell/ml) was added and mixed well. Plates were incubated at 37°C for 24 h. Turbidity of each well was observed visually. The clear wells in the micro titer plates with the lowest concentration from each extract were considered as representing the MIC.

### Minimal Bactericidal Concentrations (MBC**)**

To determine the minimum bactericidal concentration (MBC), 10 μl of the bacterial suspension from micro titer plates from the MIC experiment was dropped onto MHA in triplicate to observe viability following aerobic incubation at 37°C for 24 h. The MBC was read by determining the lowest concentration of the bacterial extracts that reduced the viability of the initial bacterial inoculum by ≥99.9%.

### HPLC-MS Analysis of Bacterial Extracts

For the analysis of natural products present in the ethyl acetate extract, the extracts were evaporated to dryness and dissolved in a 1/10 culture volume of methanol. HPLC-MS analysis was performed using a Dionex Ultimate 3000 system coupled to a Bruker AmaZon X mass spectrometer and an Acquity UPLC BEH C18 1.7 μm RP column (Waters) with an acetonitrile (0.1% formic acid) in H_2_O (0.1% formic acid) gradient ranging from 5 to 95% over 16 min at a flow rate of 0.4 ml/min at 40°C. Chromatograms were analyzed using Bruker Compass Data Analysis 4.3.

## Results

### Isolation of EPNs

**FIGURE 1:**
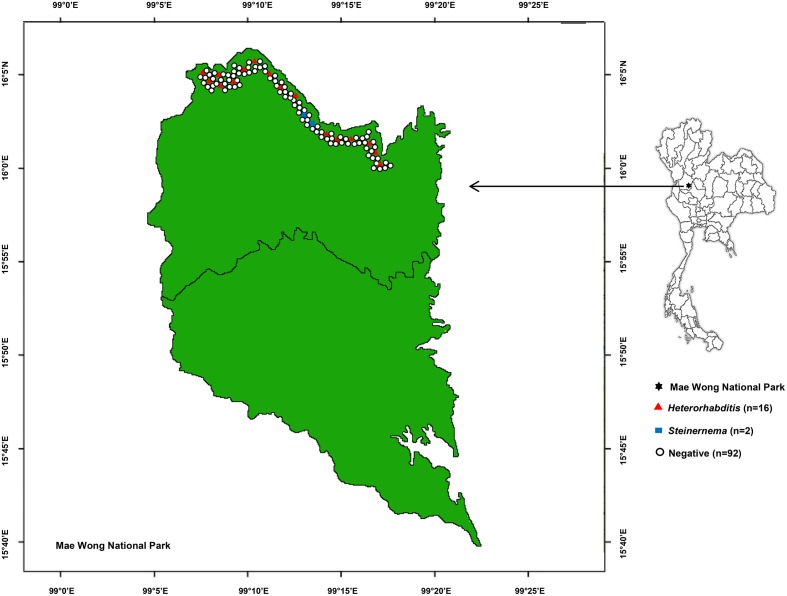
Map of 110 soil sampling sites in Mae Wong National Park, Kamphaeng Phet Province, Thailand with positive or negative yields of *Heterorhabditis* and *Steinernema.*

A total of 550 soil samples collected from 110 locations in Mae Wong National Park, Kamphaeng Phet Province, Thailand yielded 24 EPNs (4.36**%)** belonging to *Heterorhabditis* and *Steinernema* consisting of 21 and 3 isolates, respectively (**Figure 1**). Most EPNs were isolated from loam with a soil temperature range of 14–31°C (mean 22.8°C), soil pH range of 6.0–7.0 (mean 6.7), soil moisture range of 1.0–7.5 (mean 2.0), and an above mean sea level range of 224–1275 m (mean 359.7 m).

### Identification and Phylogeny of EPNs

**FIGURE 2:**
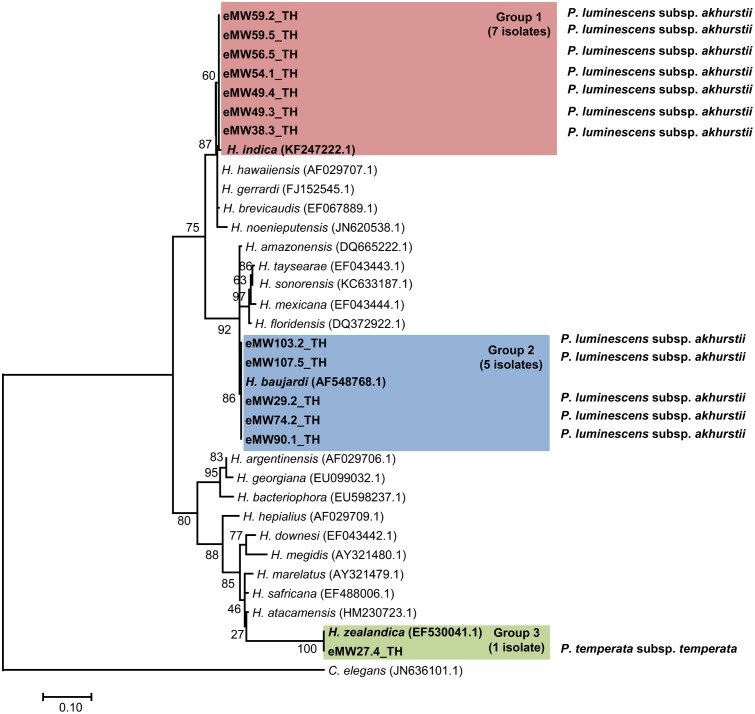
Maximum likelihood tree for *Heterorhabditis.* The phylogenetic tree was based on 634 bp of a partial region of the internal transcribed spacer (ITS) for 13 *Heterorhabditis* isolates from Mae Wong National Park, Kamphaeng Phet Province, Thailand together with 20 sequences of the *Heterorhabditis* ITS regions downloaded from NCBI. *Caenorhabditis elegans* was used as the out-group.

**FIGURE 3:**
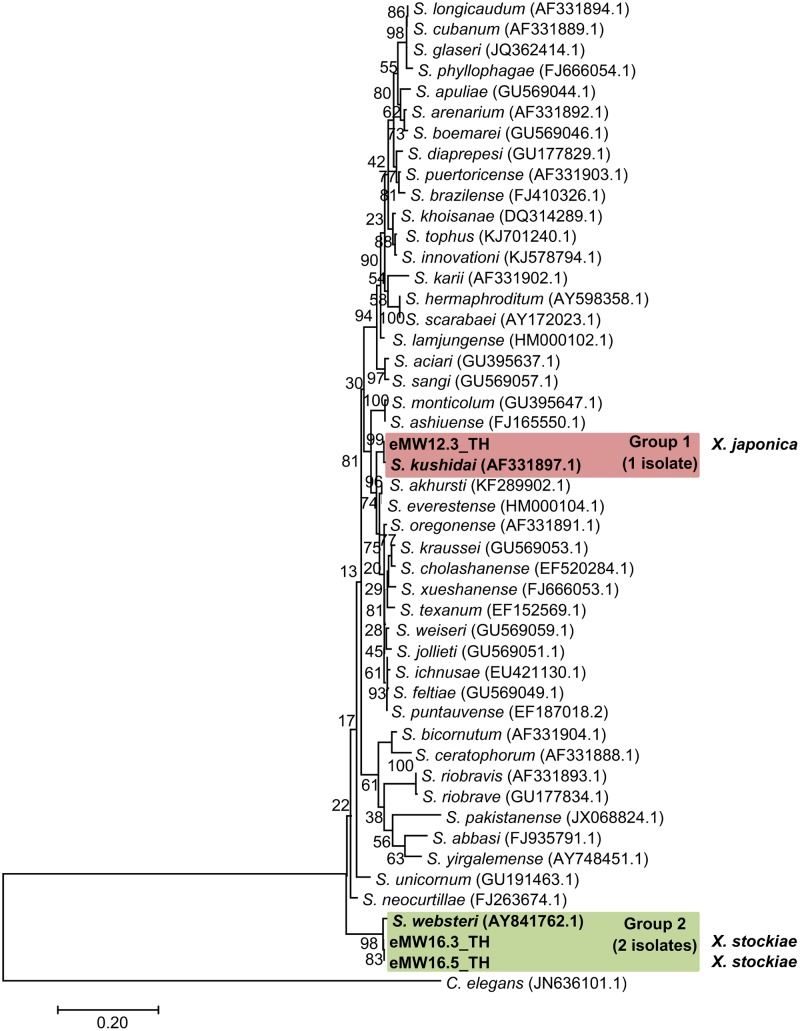
Maximum likelihood tree for *Steinernema.* The phylogenetic tree was based on 634 bp of a partial region of the *28S rRNA* gene for 3 *Steinernema* isolates from Mae Wong National Park, Kamphaeng Phet Province, Thailand together with 44 sequences of a region of the *Steinernema 28S rRNA* gene downloaded from NCBI. *C. elegans* was used as the out-group.

In total, 16 EPN isolates were identified by BLASTN analysis of sequence obtained from a region of the *28S rRNA* gene for *Steinernema* and from the ITS locus for *Heterorhabditis*. Genomic DNA could not be obtained from the remaining eight isolates owing to contamination by protozoa and fungi.We identified 13 *Heterorhabditis* isolates as *H. indica* (seven isolates with 99% identity), *H. baujardi* (Five isolates with 99% identity), and *H. zealandica* (one isolate with 100% identity). Phylogenetic tree analysis of *Heterorhabditis* isolates (Accession No. KY471364–KY471376) obtained in the present study (13 isolates) and *Heterorhabditis* (20 identified species downloaded from the NCBI database) revealed 3 main groups: group 1 consisted of an isolate of *H. indica* (KF247222.1) and 7 isolates of *Heterorhabditis* from the present study; group 2 contained *H. baujardi* (AF548768.1) and 5 isolates of *Heterorhabditis*; and group 3 consisted of one isolate of *H. zealandica* (EF530041.1) and one isolate of *Heterorhabditis* from our study (**Figure 2**). In addition, the 3 isolates of *Steinernema* (Accession No. KY454617–KY454619) were identified as *S. websteri* (2 isolates, 99% identity) and *S. kushidai* (1 isolate, 99% identity). The former were closely related to *S. websteri* (AY841762.1) and the latter was most closely related to *S. kushidai* (AF331897.1) (**Figure 3**).

### Identification and Phylogeny of *Photorhabdus* and *Xenorhabdus*

**FIGURE 4:**
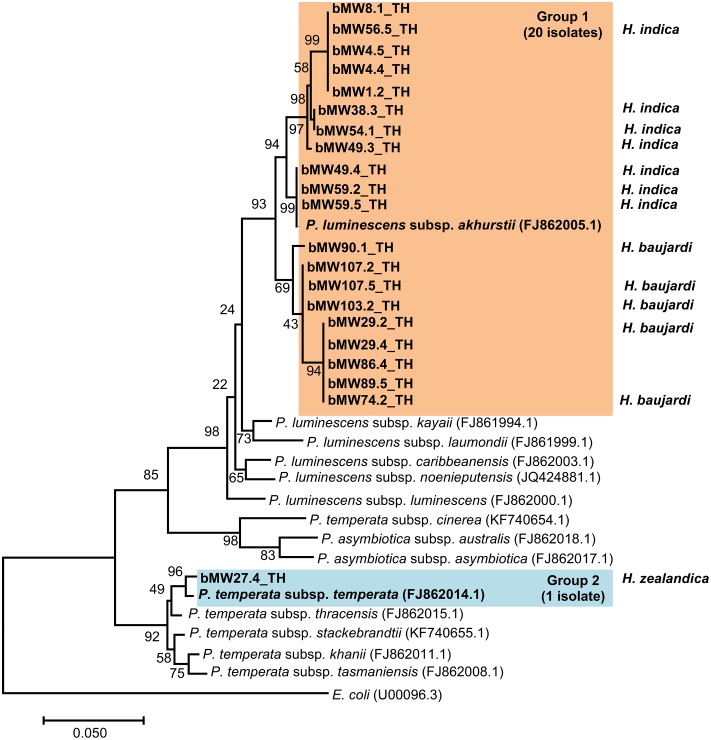
Maximum likelihood tree for *Photorhabdus.* The phylogenetic tree was based on 588 bp of the partial *rec*A gene for 21 *Photorhabdus* isolated from Mae Wong National Park, Kamphaeng Phet Province, Thailand together with 14 sequences of the *Photorhabdus rec*A gene downloaded from NCBI.

**FIGURE 5:**
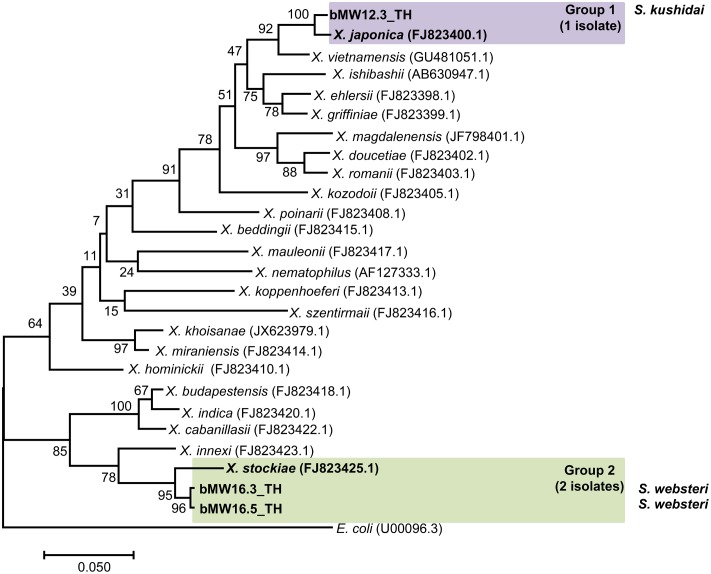
Maximum likelihood tree for *Xenorhabdus.* The phylogenetic tree was based on a 588 bp region of *rec*A for 3 *Xenorhabdus* isolates from Mae Wong National Park, Kamphaeng Phet Province, Thailand together with 23 sequences of the *Xenorhabdus rec*A gene downloaded from NCBI.

Preliminary identification of symbiotic bacteria at the genus level was based on colony morphology on NBTA agar. In total, 24 EPNs presented as *Photorhabdus* (green colony) on NBTA agar (21 isolates) and *Xenorhabdus* (blue colony) on NBTA agar (3 isolates). BLASTN analysis of 588 bp *rec*A sequences revealed that 20 isolates of *P. luminescens* subsp. *akhurstii* and 1 isolate of *P. temperata* subsp. *temperata* (Accession No. KY436903–KY436923) could be identified with 97–99% identity. Phylogenetic tree analysis of the 21 *Photorhabdus* isolates demonstrated 2 distinct groups. Group 1 was composed of 20 isolates that were closely related to *P. luminescens* subsp. *akhurstii* (FJ862005.1) and group 2 contained only a single isolate that was related to *P. temperata* subsp. *temperata* (FJ862014.0) (**Figure 4**). In turn, the three isolates of *Xenorhabdus* (Accession No. KY404049–KY404051) were identified as *Xenorhabdus stockiae* (two isolates) with 97% identity and *X. japonica* (one isolate) with 98% similarity. **Figure 5** shows the phylogenetic tree of *Xenorhabdus*, which was divided into two groups. Group 1 contained only one study isolate and a *rec*A sequence of *X. japonica* (FJ823400.1). Group 2 included two study isolates that were closely related to *X*. *stockiae* (FJ823425.1).

### Antimicrobial Activity

**FIGURE 6:**
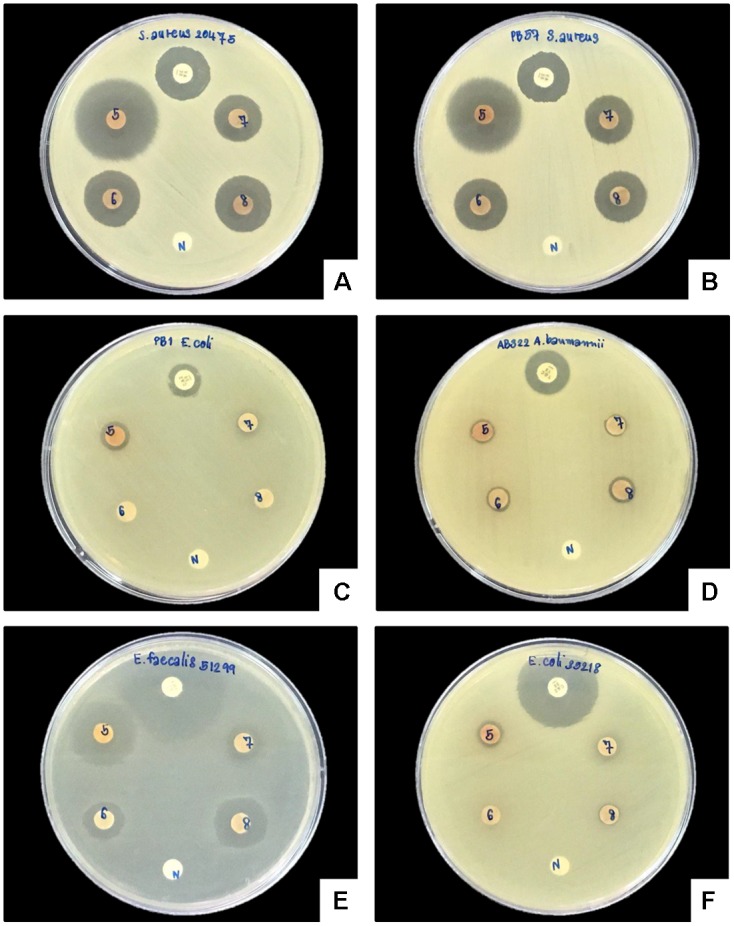
Disk diffusion method of bacterial extracts against six drug resistant bacteria. Clear zone of *S. aureus* ATCC^®^ 20475 **(A)**, PB57 *S. aureus* (MRSA; **B**), PB1 *E. coli* (ESBL+MDR; **C**), AB322 *A. baumannii* (MDR; **D**), *E. faecalis* ATCC^®^ 51299 **(E)**, and *E. coli* ATCC^®^ 35218 **(F)** after exposure to bacterial extracts from bMW27.4_TH *P. temperata* subsp. *temperata* (5), bMW49.3_TH *P. luminescens* subsp. *akhurstii* (6), bMW56.5_TH *P. luminescens* subsp. *akhurstii* (7), bMW59.2_TH *P. luminescens* subsp. *akhurstii* (8), Antibiotic disks (P) and Negative control (N).

**FIGURE 7:**
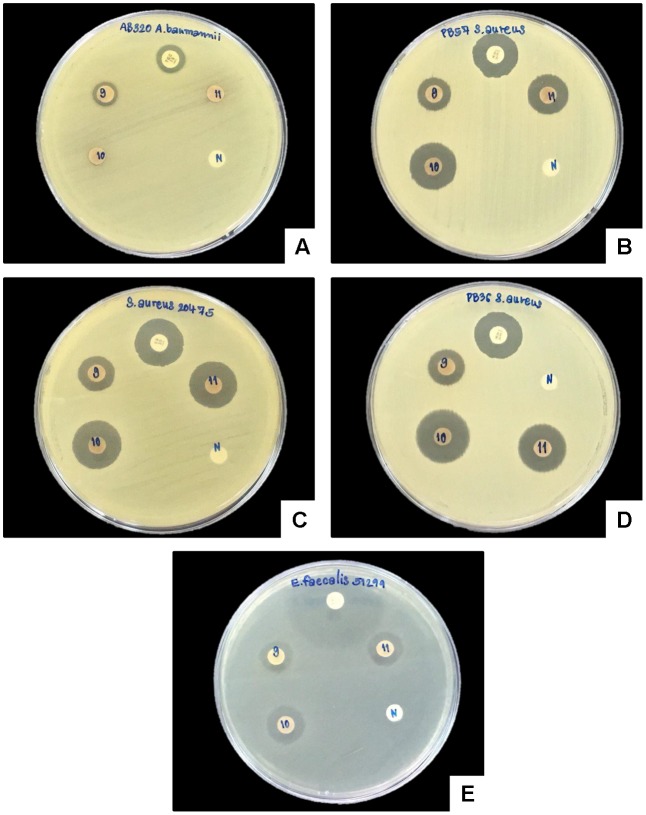
Disk diffusion method of bacterial extracts against five drug resistant bacteria. Clear zone of AB320 *A. baumannii* (XDR; **A**), PB57 *S. aureus* (MRSA; **B**), *S. aureus* ATCC^®^ 20475 **(C)**, PB- *S. aureus* (MRSA; **D**) and *E. faecalis* ATCC^®^ 51299 **(E)** after exposure to bacterial extracts from bMW59.5_TH *P. luminescens* subsp. *akhurstii* (9), bMW90.1_TH *P. Luminescens* subsp. *akhurstii* (10), bMW103.2_TH *P. luminescens* subsp. *akhurstii* (11), Antibiotic disks (P) and Negative control (N).

A clear zone indicating growth inhibition of drug resistant bacteria was demonstrated after exposure to the bacterial extraction from 2 isolates of *Xenorhabdus* and nine isolates of *Photorhabdus* (**Figures 6**, **7** and **Table 1**). The active isolates were *P. luminescens* subsp. *akhurstii* (bMW1.2_TH, bMW8.1_TH, bMW49.3_TH, bMW56.5_TH, bMW59.2_TH, bMW59.5_TH, bMW90.1_TH, and bMW103.2_TH along with *P. temperata* subsp. *temperata* bMW27.4_TH). The most potent *Photorhabdus* isolate was bMW27.4_TH *P. temperata* subsp. *temperata*, which could inhibit up to 10 strains of drug resistant bacteria AB320 *A. baumannii* (extensively drug resistant or XDR), AB321 *A. baumannii* (mutidrug resistant or MDR), AB322 *A. baumannii* (MDR), *S. aureus* ATCC^®^ 20475, PB36 *S. aureus* (methicillin-resistant *S. aureus* or MRSA*)*, PB57 *S. aureus* (MRSA), *E. coli* ATCC^®^ 35218, PB1 *E. coli* (ESBL+MDR), PB30 *P. aeruginosa* (MDR), and *E. faecalis* ATCC^®^ 51299. All *Photorhabdus* isolates could inhibit *S. aureus* ATCC^®^ 20475, PB36 *S. aureus* (MRSA), and PB57 *S. aureus* (MRSA). In addition, the bacterial extraction from bMW27.4_TH *P. temperata* subsp. *temperata* could inhibit the growth of *S. aureus* ATCC^®^ 20475 with a clear zone larger than 25 mm. However, both isolates of *X. stockiae* (bMW16.3_TH and bMW16.5_TH) inhibited only the growth of *P. aeruginosa*.

**Table 1:** Antibacterial activity of *Photorhabdus* extracts against drug resistant bacteria as assessed by disk diffusion.

Bacteria list (code)	Ability to inhibit the growth of drug resistant bacteria											


	AB320 *A. baumannii* (XDR)^a^	AB321 *A. baumannii* (MDR)^b^	AB322 *A. baumannii* (MDR)^b^	*S. aureus* ATCC^®^ 20475 (MRSA)^c^	PB36 *S. aureus* (MRSA)^c^	PB57 *S. aureus* (MRSA)^c^	*E. coli* ATCC^®^ 35218	PB1 *E. coli* (ESBL+ MDR)^b^*^,^*^d^	*P. aeruginosa* ATCC^®^ 27853	PB30 *P. aeruginosa* (MDR)^b^	*E. faecalis* ATCC^®^ 51299	*K. pneumonia* ATCC^®^ 700603
*X. stockiae* (bMW16.3_TH)	-	-	-	-	-	-	-	-	++	-	-	-
*X. stockiae* (bMW16.5_TH)	-	-	-	-	-	-	-	-	+	-	-	-
*P. luminescens* subsp. *akhurstii* (bMW1.2_TH)	-	+	-	++	++	++	-	-	+	-	+	-
*P. luminescens* subsp. *akhurstii* (bMW8.1_TH)	-	-	-	++	++	++	-	-	+	-	+	-
*P. temperata* subsp. *temperata* (bMW27.4_TH)	+	+	+	++	++	++	+	+	-	+	+	-
*P. luminescens* subsp. *akhurstii* (bMW49.3_TH)	-	+	+	++	++	++	-	-	-	-	+	-
*P. luminescens* subsp. *akhurstii* (bMW56.5_TH)	+	+	+	++	++	++	-	-	+	-	+	-
*P. luminescens* subsp. *akhurstii* (bMW59.2_TH)	+	+	+	++	++	++	-	-	+	-	+	-
*P. luminescens* subsp. *akhurstii* (bMW59.5_TH)	+	-	+	++	++	++	-	+	+	-	+	+
*P. luminescens* subsp. *akhurstii* (bMW90.1_TH)	-	-	-	++	++	++	-	-	+	-	+	-
*P. luminescens* subsp. *akhurstii* (bMW103.2_TH)	-	-	-	++	++	++	-	-	-	-	+	-

*-No inhibition, + a zone of poor inhibition, ++ a zone of larger inhibition, ^a^Extensively drug resistant, ^b^Multidrug resistant, ^c^Methicillin resistant *Staphylococcus aureus*, and ^d^Extended spectrum beta-lactamase*.

Based on MIC and MBC testing, eight bacterial extracts from all *P. luminescens* subsp. *akhurstii* isolates were evaluated against three strains of drug resistant bacteria including *S. aureus* ATCC^®^ 20475, PB36 *S. aureus* (MRSA), and PB57 *S. aureus* (MRSA). In addition, one bacterial extract from a *P. temperata* subsp. *temperata* isolate was evaluated against five strains of drug resistant bacteria including *S. aureus* ATCC^®^ 20475, PB36 *S. aureus* (MRSA), PB57 *S. aureus* (MRSA), *E. coli* ATCC^®^ 35218, and PB30 *P. aeruginosa* (MDR). The growth of drug resistant bacteria was inhibited by *Photorhabdus* extracts with MICs ranging from 7.81 to 0.86 mg/ml. PB36 *S. aureus* (MRSA) was the most susceptible to all extracts with MICs ranging from 1.95 to 0.86 mg/ml (**Table 2**) and MBCs ranging from 0.86 to 15.625 mg/ml (Supplementary Figures S1–S4).

**Table 2:** Antibacterial activity of *Photorhabdus* extracts against drug resistant bacteria as assessed by minimal inhibitory concentration and minimal bactericidal concentration (mg/ml).

Bacteria list (Code)	Concentration of inhibition (mg/ml)									
	*S. aureus* ATCC^®^ 20475 (MRSA)		PB36 *S. aureus* (MRSA)		PB57 *S. aureus* (MRSA)		*E. coli* ATCC^®^ 35218		PB30 *P. aeruginosa* (MDR)	
	MIC	MBC	MIC	MBC	MIC	MBC	MIC	MBC	MIC	MBC
*P. luminescens* subsp. *akhurstii* (bMW1.2_TH)	1.95	1.95	1.95	1.95	3.90	3.90	ND	ND	ND	ND
*P. luminescens* subsp. *akhurstii* (bMW8.1_TH)	1.95	1.95	1.95	1.95	3.125	3.125	ND	ND	ND	ND
*P. temperata* subsp. *temperata* (bMW27.4_TH)	0.86	0.86	0.86	0.86	0.86	0.86	1.718	1.718	1.718	3.44
*P. luminescens* subsp. *akhurstii* (bMW49.3_TH)	1.95	1.95	1.95	1.95	1.95	0.98	ND	ND	ND	ND
*P. luminescens* subsp. *akhurstii* (bMW56.5_TH)	3.90	3.90	1.95	1.95	3.90	3.90	ND	ND	ND	ND
*P. luminescens* subsp. *akhurstii* (bMW59.2_TH)	3.90	7.81	1.95	1.95	3.90	3.90	ND	ND	ND	ND
*P. luminescens* subsp. *akhurstii* (bMW59.5_TH)	1.95	1.95	1.95	1.95	3.125	3.125	ND	ND	ND	ND
*P. luminescens* subsp. *akhurstii* (bMW90.1_TH)	7.81	7.81	1.95	1.95	1.95	3.90	ND	ND	ND	ND
*P. luminescens* subsp. *akhurstii* (bMW103.2_TH)	3.90	15.625	1.95	1.95	3.90	3.90	ND	ND	ND	ND

*ND, not determined*.

### HPLC-MS Analysis

Ethyl acetate extracts were checked by HPLC-MS for known compound classes (Supplementary Figure S5 and Table S2). All isolates of *Xenorhabdus* and *Photorhabdus* produced GameXPeptide derivatives. All of the *Photorhabdus* spp. produced isopropylstilbene and all *Xenorhabdus* isolates produced xenoamicin derivatives. Six isolates of *P. luminescens* subsp. *akhurstii* produced xenocoumacin derivatives. Additionally, mevalagmapeptide and phurealipids derivatives were found in many of the isolates (Supplementary Table S2).

## Discussion

In the present study, we surveyed soil samples in search of EPNs associated with *Xenorhabdus* and *Photorhabdus* in Mae Wong National Park, Kamphaeng Phet Province, Thailand. Our findings are consistent with previous reports (Thanwisai et al., 2012; Vitta et al., 2015, 2017), which showed that the common species of EPNs in Thailand are *H. indica* and *S. websteri* whereas *H. baujardi* represents a minor identified species, thus confirming that *H. indica* and *S. websteri* are abundant in Thailand. In addition, the current study represents the first recorded identification of *H. zealandica* and *S. kushidai* in Thailand. *H. zealandica* was first isolated from *Heteronychus arator* in Auckland, New Zealand (Poinar, 1990). *S. kushidai* was first isolated from field soil in Shizuoka Prefecture, Japan (Kushida et al., 1987). *H. zealandica* and *S. kushidai* each were identified as a single isolate in the present study, suggesting that these two species may have a low level of distribution and be restricted to near forested areas (National Parks). This conclusion is further suggested by the lack of *H. zealandica* and *S. kushidai* isolates recovered from roadside verge, fruit crops, rice fields, or the banks of rivers and ponds in Thailand in prior surveys (Thanwisai et al., 2012; Vitta et al., 2015).

Here, we reported that *X. stockiae*, *X*. *japonica*, *P. luminescens* subsp. *akhurstii*, and *P. temperata* subsp. *temperata* were dominated by *P. luminescens* subsp. *akhurstii.* This is consistent with the results of previous studies in Thailand, which recorded two species of *Xenorhabdus* including *X. stockiae* and *X. miraniensis* and two species (Five subspecies) of *Photorhabdus* including *P. luminescens* subsp. *laumondii*, *P. luminescens* subsp. *akhurstii*, *P. luminescens* subsp. *hainaensis*, *P. asymbiotica* subsp. *australis*, and *P. luminescens* subsp. *namnaonensis* throughout the country (Tailliez et al., 2010; Maneesakorn et al., 2011; Thanwisai et al., 2012; Glaeser et al., 2017). Of these, *X. miraniensis*, *P. asymbiotica* subsp. *australis*, *P. luminescens* subsp. *laumondii*, and *P. luminescens* subsp. *hainanensis* were not identified in our study. This may be due to low distribution of these bacteria. In contrast, a new record of *X*. *japonica* and *P. temperata* subsp. *temperata* in our country was established in the current study. *X*. *japonica* associated with *S. kushidai* have previously been reported from Japan (Nishimura et al., 1994); this *Steinernema*-*Xenorhabdus* complex is similar to our finding. In addition, a previous study showed that *P. temperata* subsp. *temperata* is symbiotically associated with *H. megidis* (Tailliez et al., 2010). In contrast, the current study instead demonstrated *P. temperata* subsp. *temperata* and *H. zealandica* as a novel association. *H. zealandica* from South Africa has been previously reported to be symbiotic with *Photorhabdus heterorhabditis* (Ferreira et al., 2014), which suggests that *P. temperata* subsp. *temperata* manifests a broad host range with *Heterorhabditis* spp.

Antibiotics have the potential to prevent or treat infectious agents that can cause patient morbidity and mortality. Accordingly, the development of antibiotic resistance in infectious bacteria is of substantial concern. Our study demonstrated that crude extracts from *P. temperata* subsp. *temperata* and *P. luminescens* subsp. *akhurstii* showed good antimicrobial activity against drug resistant bacteria. The most common bacteria exhibiting susceptibility to *Photorhabdus* extract were *S. aureus* ATCC^®^ 20475, PB36 *S. aureus* (MRSA), and PB57 *S. aureus* (MRSA). This may be due to the ability of *Photorhabdus* to produce several secondary metabolites including insecticidal compounds and antimicrobials such as isopropylstilbene, ethylstilbene, epoxystilbene, photobactin, and ulbactin E (Bode, 2009). In support of the antibacterial activity of this bacterium, *P. temperata* showed many bioactivities, e.g., insecticidal, antioxidant, and antibacterial activities (Jang et al., 2012; Ullah et al., 2014, 2015). Phthalic acid or 1, 2-benzenedicarboxylic acid purified from *P. temperata* M1021 showed antibacterial activity with MIC values ranged between 0.1 and 0.5 M (Ullah et al., 2014). In addition, benzaldehyde purified from *P. temperata* M1021 showed antibacterial activity with MIC values ranging between 6 and 10 mM and antifungal activity with MIC values between 8 and 10 mM (Ullah et al., 2015).

Notably, in the present study *P. temperata* subsp. *temperata* could inhibit the growth of 10 strains of drug resistant bacteria, consistent with previous reports that this bacterium inhibited the growth of *Salmonella* Typhimurium KCTC 1926 and *Micrococcus luteus* KACC 10488 (Jang et al., 2012). This confirmed that *P. temperata* is the broad activity against bacterial pathogens. *P. temperata* may therefore serve as a suitable alternative choice for application as a biocontrol agent in drug industries. In addition, this indicates that several antimicrobial compounds or effective secondary metabolites may be produced by this bacterium that might be used for the production of broad spectrum antimicrobial agents.

Furthermore, several isolates of *P. luminescens* subsp. *akhurstii* also exhibited good bactericidal activity in the current study against several strains of drug resistant bacteria. *P. luminescens* subsp. *akhurstii* previously showed nematicidal and antibacterial activities (Sharma et al., 2002; Qiu et al., 2009, 2016). Lumicins, novel bacteriocins produced by *P. luminescens* subsp. *akhurstii* strain W14, showed bioactivity against other *Photorhabdus* and *E. coli* (Sharma et al., 2002). The current findings thus represent a starting point for understanding the antimicrobial activity of *Photorhabdus*. Further research related to the facilities of *Photorhabdus* and identifying the active compounds in the extract from. *Photorhabdus* culture would likely be valuable to facilitate the control of infectious disease in an effective and sustainable manner.

In comparison, *X. stockiae*, a symbiotic bacterium found abundantly in Thailand, appears to have less bactericidal effects against drug-resistant bacteria. Previous studies show that *X. stockiae* and their secreted products have good effects against mushroom mites and bacteria that cause mastitis in cows such as *S. aureus*, *S. intermedius*, *Streptococcus agalactiae*, *E. faecalis*, *K. pneumoniae*, and *E. coli* (Bussaman and Rattanasena, 2016; Namsena et al., 2016). In addition, nanoparticles of extracellular metabolites from *X. stockiae* (KT835471) showed bactericidal effect against six different pathogens (Chandrakasan et al., 2017).

Overall, based on the MIC and MBC of *Xenorhabdus* and *Photorhabdus* against drug resistant bacteria, we found that different species and isolates of these bacteria gave variable effects on different species and strains of drug resistant bacteria. This may arise because of the ability of each symbiotic bacteria to produce effective metabolites or the susceptibility of drug resistant bacteria to the respective metabolites produced by each symbiote.

Based on the analysis of ethyl acetate extracts by HPLC-MS, all isolates produced GameXPeptide derivatives, a compound known to be common among the genera but with an unknown biological activity (Bode et al., 2012; Nollmann et al., 2015a). Six isolates produced xenocoumacin derivatives (Reimer et al., 2009) or amicoumacin derivatives (Park et al., 2016) being potent antibiotics, while all of the *Photorhabdus* spp. produced isopropylstilbene that has multiple biological activities including antibiotic activity (Li et al., 1995; Buscató et al., 2013). All *Xenorhabdus* isolates produced xenoamicin derivatives showing weak antiprotozoal activity (Zhou et al., 2013). Additionally, we identified mevalagmapeptide (Bode et al., 2015, 2012) and the juvenile hormone epoxide hydrolase inhibitors phurealipids (Nollmann et al., 2015b) in many of the isolates.

## Conclusion

We have extended the basic knowledge regarding EPNs and their symbiotic bacteria, *Xenorhabdus* and *Photorhabdus*, in Mae Wong National Park of Thailand. The common symbiotic species in the study area are *H. indica* associated with *P. luminescens* subsp. *akhurstii* and *S. websteri* associated with *X. stockiae. H. zealandica*, and *S. kushidai* represent newly recorded EPNs in Thailand with low distribution. *X*. *japonica* and *P. temperata* subsp. *temperata* reflect new observations of symbiotic bacteria in Thailand. Furthermore, the association between *P. temperata* subsp. *temperata* and *H. zealandica* has not been previously reported in any location. Based on MIC and MBC, all isolates of *Xenorhabdus* and *Photorhabdus* have bactericidal activity with variable effect on different species or strains of drug resistant bacteria. Nevertheless, *P. temperata* subsp. *temperata* represents the best symbiotic bacteria for inhibiting the growth of AB320 *A. baumannii* (extensively drug resistant or XDR), AB321 *A. baumannii* (multidrug resistant or MDR), AB322 *A. baumannii* (MDR), *S. aureus* ATCC^®^ 20475, PB36 *S. aureus* (methicillin-resistant *S. aureus* or MRSA*)*, PB57 *S. aureus* (MRSA*)*, *E. coli* ATCC^®^ 35218, PB1 *E. coli* (ESBL+MDR*)*, PB30 *P. aeruginosa* (MDR), and *E. faecalis* ATCC^®^ 51299. *P. temperata* subsp. *temperata* may be of interest for further study regarding its antimicrobial activity. Our findings enhance the understanding of the distribution of EPN-bacteria complexes and provide a foundation for subsequent research toward identifying potential antimicrobial compounds that may represent effective and sustainable resources for combating drug resistant microbial infections.

## Author Contributions

Conceived and designed the experiments: AT, AV, SS, and NC. Performed the experiments: PM, TY, CF, MS, and THY. Analyzed the data: AT, AV, and PM. Contributed reagents/materials/analysis tools: AT, AV, SS, and NC. Chemical analysis of bacterial extracts: NT and HB. Wrote the paper: PM, AT, AV, SS, and HB.

## Conflict of Interest Statement

The authors declare that the research was conducted in the absence of any commercial or financial relationships that could be construed as a potential conflict of interest.
