# Supplementary material for: Screening of the Antimicrobial Activity against Drug Resistant Bacteria of Photorhabdus and Xenorhabdus Associated with Entomopathogenic Nematodes from Mae Wong National Park, Thailand
Source: Front Microbiol. 2017 Jun 28;8:1142. doi: 10.3389/fmicb.2017.01142 (PMC5487437; doi:10.3389/fmicb.2017.01142)
Supplement: Supplementary file 1 [file Table_1.docx]

Supplementary Table 1. List of clinical isolates of drug resistant bacteria tested

| Date of specimen collection | strain | specimen | Pattern of antibiotic resistance |
| --- | --- | --- | --- |
| 02/14/11 | AB-320 *A. baumannii* | sputum | XDR^a^ |
| 02/14/11 | AB-321 *A. baumannii* | urine | MDR^b^ |
| 02/14/11 | AB-322 *A. baumannii* | sputum | MDR^b^ |
| 12/21/14 | PB36 *S. aureus* | sputum | MRSA^c^ |
| 12/23/14 | PB57 *S. aureus* | blood | MRSA^c^ |
| 12/15/14 | PB1 *E. coli* | urine | ESBL+MDR^b,d^ |
| 12/20/14 | PB30 *P. aeruginosa* | sputum | MDR^b^ |

^a^Extensively drug resistant, ^b^ Multidrug resistant, ^c^ Methicillin resistant *Staphylococcus aureus*, and ^d^Extended spectrum beta-lactamase

Supplementary Table 2. Summary of strains and the known compounds that they produce in ethyl acetate extracts after 72 hours of growth

| **Strain number** | **Species** | **GameXPeptide derivatives** | **Isopropylstilbene** | **Xenoamicin derivatives** | **Xenocoumacin derivatives** | **Mevalagmapeptide derivatives** | **Phurealipid derivatives** |
| --- | --- | --- | --- | --- | --- | --- | --- |
| MW59.5 | *Photorhabdus luminescens* subsp. *akhurstii* | + | + | - | + | - | - |
| MW49.3 | *Photorhabdus luminescens* subsp. *akhurstii* | + | + | - | + | + | - |
| MW8.1 | *Photorhabdus luminescens* subsp. *akhurstii* | + | + | - | - | + | + |
| MW27.4 | *Photorhabdus temperata* subsp. *temperata* | + | + | - | - | - | + |
| MW103.2 | *Photorhabdus luminescens* subsp. *akhurstii* | + | + | - | + | + | - |
| MW56.5 | *Photorhabdus luminescens* subsp. *akhurstii* | + | + | - | + | - | + |
| MW59.2 | *Photorhabdus luminescens* subsp. *akhurstii* | + | + | - | + | + | - |
| MW1.2 | *Photorhabdus luminescens* subsp. *akhurstii* | + | + | - | + | + | + |
| MW16.3 | *Xenorhabdus stockiae* | + | - | + | - | + | - |
| MW16.5 | *Xenorhabdus stockiae* | + | - | + | - | + | - |
