# Supplementary material for: Screening of the Antimicrobial Activity against Drug Resistant Bacteria of Photorhabdus and Xenorhabdus Associated with Entomopathogenic Nematodes from Mae Wong National Park, Thailand
Source: Front Microbiol. 2017 Jun 28;8:1142. doi: 10.3389/fmicb.2017.01142 (PMC5487437; doi:10.3389/fmicb.2017.01142)
Supplement: Supplementary file 2 [file Image_1.PDF]

Supplementary Figure S1, S2, S3, S4 Minimum Bactericidal Concentration (MBC)

PB 36 *S. aureus*

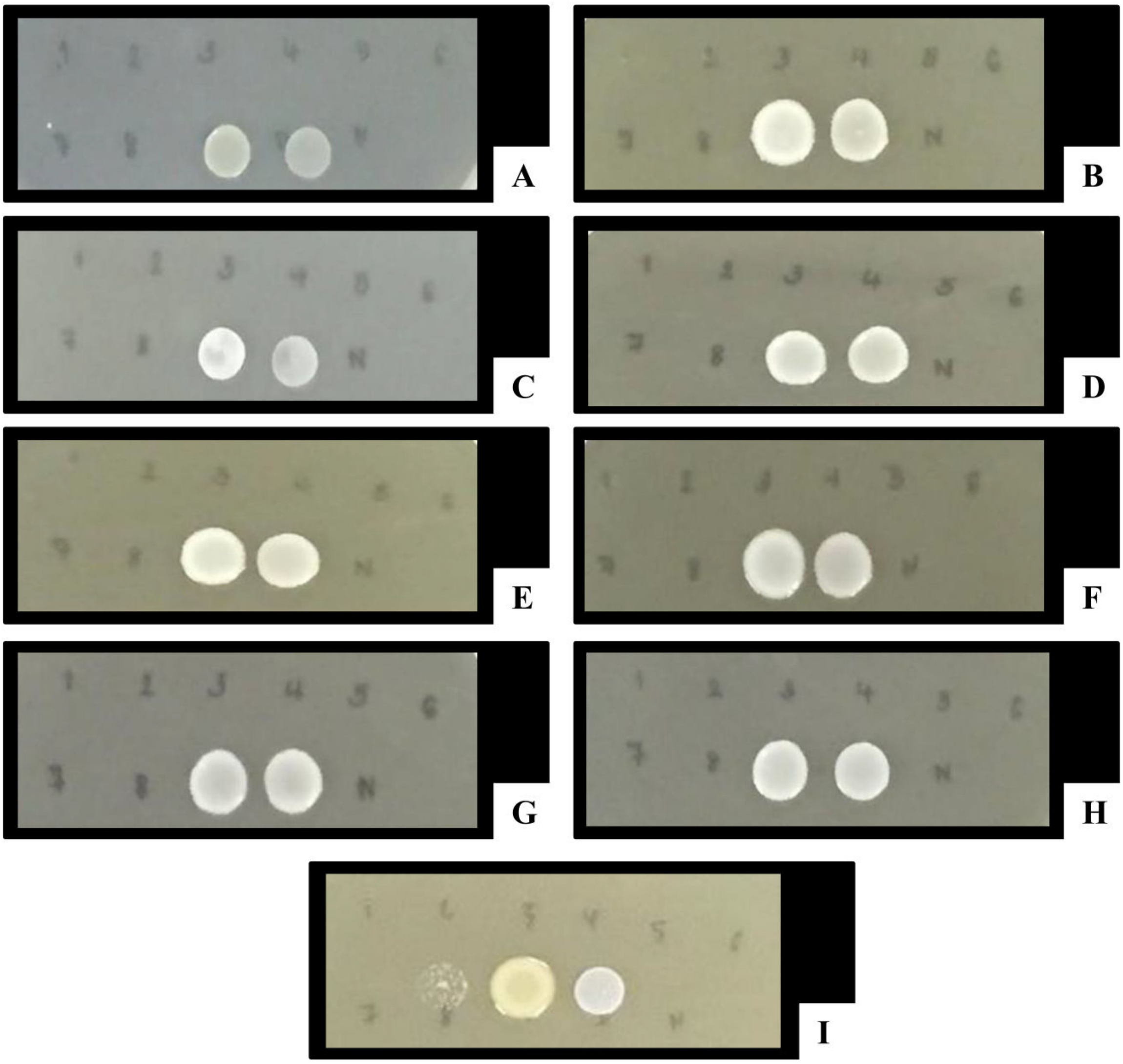

Supplementary Figure S1 MBC of PB36 *S. aureus* (MRSA) after exposure to bacterial extracts from *P. luminescens* subsp. *akhurstii* (bMW1.2_TH, bMW8.1_TH, bMW49.3_TH, bMW56.5_TH, bMW59.2_TH, bMW59.5_TH, bMW90.1_TH, bMW103.2_TH **(A-H)** and *P. temperata* subsp. *temperata* (bMW27.4_TH) **(I)**.

PB 57 *S. aureus*

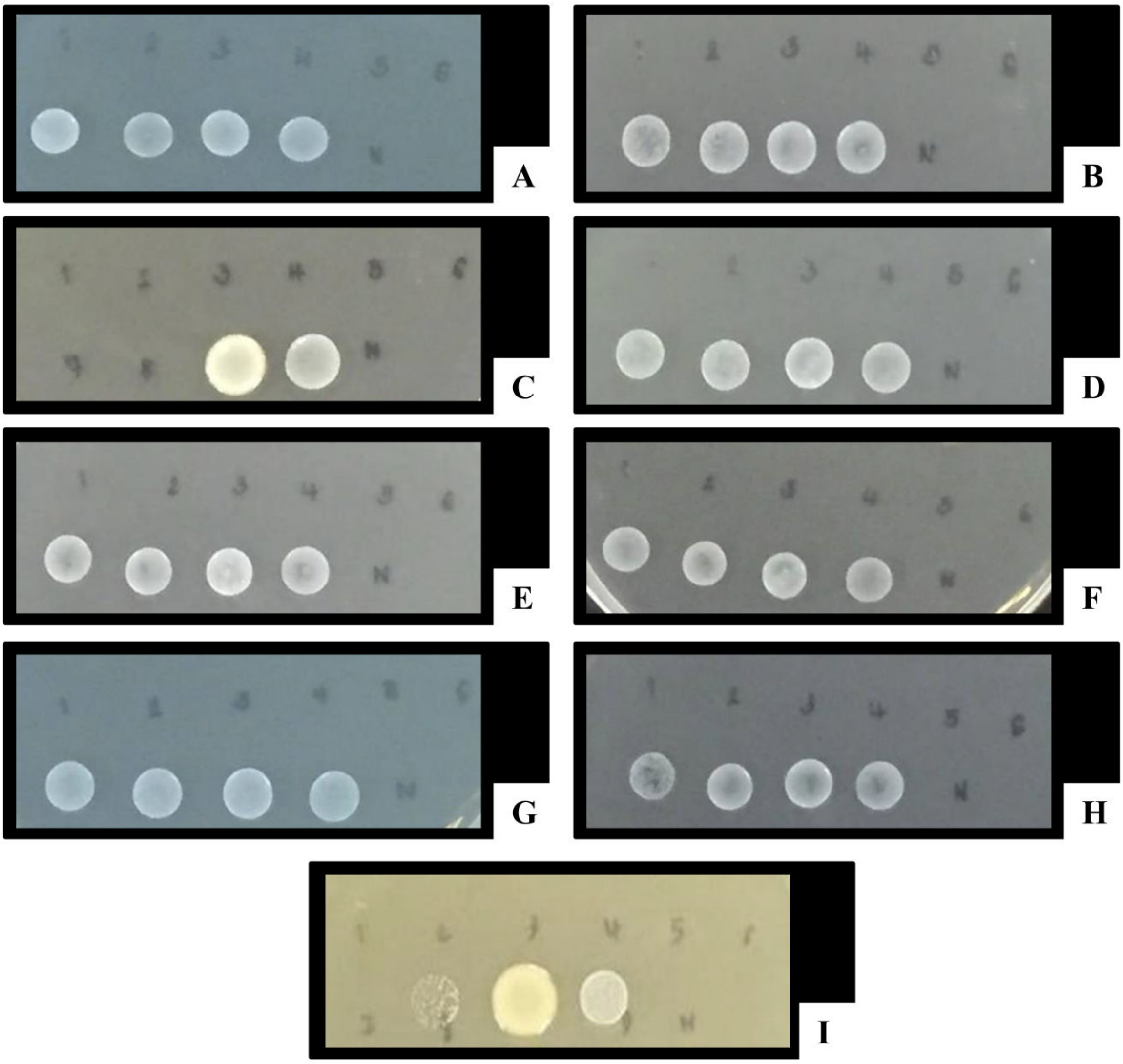

Supplementary Figure S2 MBC of PB57 *S. aureus* (MRSA) after exposure to bacterial extracts from *P. luminescens* subsp. *akhurstii* (bMW1.2_TH, bMW8.1_TH, bMW49.3_TH, bMW56.5_TH, bMW59.2_TH, bMW59.5_TH, bMW90.1_TH, bMW103.2_TH **(A-H)** and *P. temperata* subsp. *temperata* (bMW27.4_TH) **(I)**.

***S. aureus* ATCC® 20475**

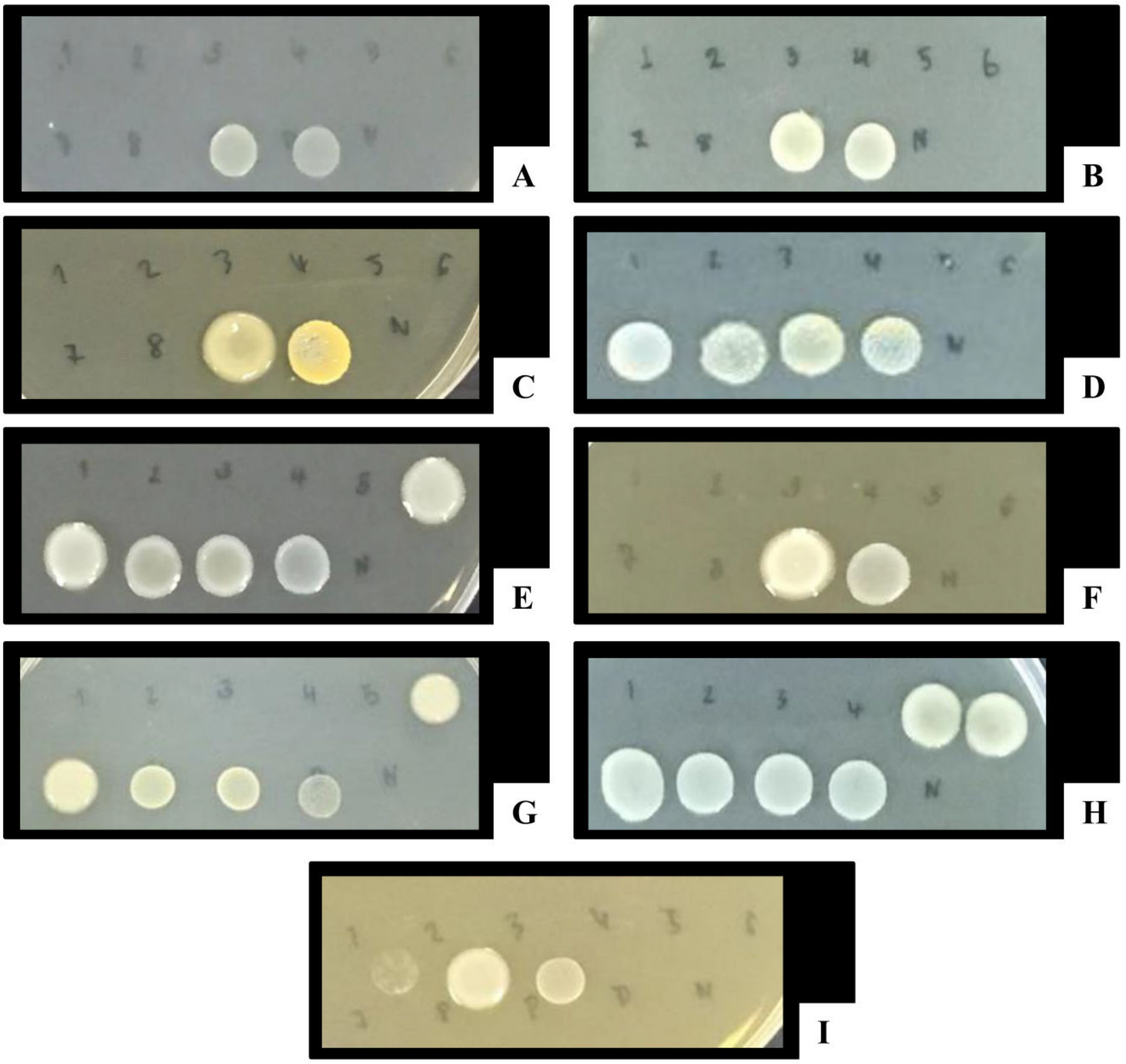

Supplementary Figure S3 MBC of *S. aureus* ATCC® 20475 after exposure to bacterial extracts from *P. luminescens* subsp. *akhurstii* (bMW1.2_TH, bMW8.1_TH, bMW49.3_TH, bMW56.5_TH, bMW59.2_TH, bMW59.5_TH, bMW90.1_TH, bMW103.2_TH **(A-H)** and *P. temperata* subsp. *temperata* (bMW27.4_TH) **(I)**.

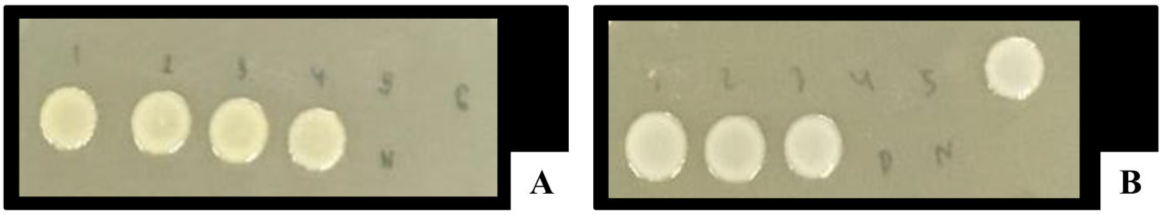

Supplementary Figure S4 MBC of *E. coli* ATCC® 35218 **(A)** and PB30 *P. aeruginosa* (MDR) **(B)** after exposure to bacterial extracts from *P. temperata* subsp. *temperata* (bMW27.4_TH).

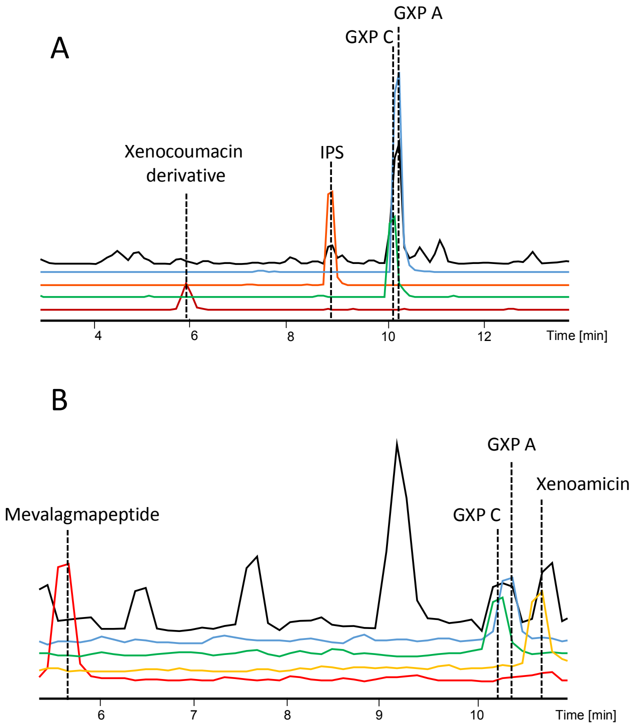

Supplementary Figure S5 Base peak chromatograms (black) of representative extracts of **A.** *Photorhabdus luminescens* MW59.5 and **B.** *Xenorhabdus stockiae* MW16.3. Shown are extracted ion chromatograms at five times magnification of two derivatives of GameXPeptide (GXP A: blue, GXP C: green (Bode et al., 2012; Nollmann et al., 2015a), isopropylstilbene (IPS: orange (Li et al., 1995), a xenocoumacin derivative (dark red)(Park et al., 2016; Reimer et al., 2009), xenoamicin (gold) (Zhou et al., 2013) and mevalagmapeptide (red)(Bode et al., 2015; 2012).
